# Paper-Based Aptasensors: Working Principles, Detection Modes, and Applications

**DOI:** 10.3390/s23187786

**Published:** 2023-09-10

**Authors:** Anastasios Economou, Christos Kokkinos, Leda Bousiakou, Tibor Hianik

**Affiliations:** 1 Laboratory of Analytical Chemistry, Department of Chemistry, National and Kapodistrian University of Athens, 15771 Athens, Greece; christok@chem.uoa.gr; 2 IMD Laboratories Co., R&D Section, Lefkippos Technology Park, National Centre for Scientific Research (NCSR) Demokritos, Agia Paraskevi, P.O. Box 60037, 15130 Athens, Greece; leda@imdlaboratories.gr; 3 Department of Nuclear Physics and Biophysics, Faculty of Mathematics, Physics and Informatics, Comenius University, Mlynská dolina F1, 84248 Bratislava, Slovakia; tibor.hianik@fmph.uniba.sk

**Keywords:** aptasensors, aptamers, paper-based devices, biosensors

## Abstract

Aptamers are short oligonucleotides designed to possess high binding affinity towards specific target compounds (ions, molecules, or cells). Due to their function and unique advantages, aptamers are considered viable alternatives to antibodies as biorecognition elements in bioassays and biosensors. On the other hand, paper-based devices (PADs) have emerged as a promising and powerful technology for the fabrication of low-cost analytical tools, mainly intended for on-site and point-of-care applications. The present work aims to provide a comprehensive overview of paper-based aptasensors. The review describes the fabrication methods and working principles of paper-based devices, the properties of aptamers as bioreceptors, the different modes of detection used in conjunction with aptasensing PADs, and representative applications for the detection of ions, small molecules, proteins, and cells. The future challenges and prospects of these devices are also discussed.

## 1. Introduction

Aptamers, which are short nucleic acid sequences with high specificity for selected target compounds, were described for the first time in the early 1990s and have been coined as promising bioreceptors for analytical applications due to some distinct advantages over antibodies, including low cost, high stability, easy synthesis, and a wide range of targets [1]. Over the last few years, aptamers have found wide application in different fields such as clinical diagnostics, environmental analysis, food control, and pharmaceutical analysis [2,3,4,5]. On the other hand, paper-based analytical devices (PADs) are analytical platforms using cellulose as a functional support. The field of PADs has witnessed a rapid expansion over the last decade, capitalizing on the advantageous properties of paper, including wettability, conformability, lightness, easy functionalization, and instrument-free solution flow [6].

The combination of aptamers as biorecognition elements with paper as a platform material results in versatile, low-cost, selective, sensitive, and portable analytical devices. This review aims to provide a thorough overview of paper-based aptasensors. The review describes the fabrication methods and working principles of paper-based devices, the properties of aptamers as bioreceptors, the different modes of detection used in conjunction with aptasensing PADs, and representative applications for the detection of ions, small molecules, proteins, and cells. It must be noted that the review does not cover aptamer-based lateral flow devices (LFDs) and lateral flow assays (LFAs) since these differ from PADs in that they use mainly nitrocellulose as their functional support material and are based on unidirectional flow of solutions on overlapping separate segments of (nitro)cellulose. Although LFDs and LFAs, based on aptamers as the biological recognition element, have been extensively studied and applied [7,8], the relevant literature on paper-based devices is still limited [9].

## 2. Paper-Based Devices

### 2.1. Paper as an Analytical Platform

Nowadays, paper is considered an attractive and promising platform for analytical applications. Paper consists of a network of randomly interwoven cellulose fibers in a 2-dimensional sheet format [10,11,12,13]. The fibrous and porous structure of cellulose confers some important advantages that make paper an excellent candidate as an analytical platform [14].

(1) It promotes the movement of liquids by capillary action, eliminating the need for external forces to move low volumes of samples and reagents.
(2) It is highly absorbent and offers a high surface-to-volume ratio, which enables the efficient immobilization and storage of the reagents.
(3) It enables the filtration of the sample.
(4) It is thin, light, and conformable.
(5) It is biocompatible and biodegradable.
(6) It is inexpensive and easily available worldwide.

Paper-based analytical devices (PADs) are novel analytical tools that are fabricated with cellulose as their functional substrate material and are widely used to analyze small amounts of samples [6,15]. Considering the unique properties of paper as an analytical platform, PADs represent an innovative technology for fluid handling and analysis with a wide range of applications, especially for on-site monitoring and point-of-care testing, including clinical diagnostics [16], food quality [17], pharmaceuticals [18], and environmental monitoring [19].

A milestone in the field of PADs was the seminal work of the Whitesides’ laboratory in 2007, which demonstrated that it was possible to achieve fluid flow within hydrophilic channels delimited on paper by the creation of hydrophobic barriers [20]. Paper patterning to create multidimensional flow channels actually differentiates PADs from LFAs and LFDs. This designed patterning allows the creation of directed flow within PADs [21], allowing complex sample preparation steps to be performed [22], and facilitating multiplexed detection on these devices [23].

### 2.2. Fabrication of Paper-Based Devices

The selection of the grade of paper is important for the proper operation of PADs and depends on the assay. Commercial paper grades often differ in porosity, particle retention, thickness, and flow rate, which may affect drastically the analytical and operational features of the assay. In paper-based microfluidics, most of the studies have exploited chromatographic and filter papers, which are manufactured using high-quality cotton linters with a minimum a-cellulose content of 98% and exhibiting different degrees of purity, hardness, and chemical resistance [10,11,12,14].

Two general methodologies have been applied for the fabrication of PADs: two-dimensional shaping/cutting and patterning hydrophilic/hydrophobic areas [10,11,12,13,14,24]. In the first methodology, the paper channels are generally obtained by cutting the paper manually, with an X-Y cutter/plotter, or with a CO_2_ laser cutting apparatus. Then, in most cases, the cut channels are covered with sticky tape as a backing to provide rigidity to the device. The second, and most sophisticated, method is based on creating hydrophilic patterns on paper with hydrophobic borders that define the fluidic channels.

Over the years, increasingly complex and sophisticated 2-dimensional (2D) and 3-dimensional (3D) PAD configurations (including multi-layered, vertical flow, daisy-shaped, and folding (origami)) have been proposed and implemented [25,26]. 2D PADs are fabricated on a single sheet of paper, while 3D PADs are more complicated in that they utilize multiple layers of patterned paper either in a multi-layer or a folding (origami) configuration, many of them allowing multiplexed detection [23,26].

Theoretical and practical aspects regarding the operation of PADs (such as liquid flow, flow control, surface modification, etc.) are discussed in several reviews [10,11,12,13,27,28,29].

## 3. Aptamers and Aptasensors

### 3.1. Aptamers

In the early 1990s, a new in vitro selection method was developed for nucleic acid sequences that bind their target in a highly selective manner [30,31]. This technique, termed SELEX (Systematic Evolution of Ligands by Exponential Enrichment), resulted in the discovery of aptamers. During the SELEX process, a library of random oligonucleotide sequences is exposed to the desired target. A small percentage of the library’s sequences bind to the target and is subsequently separated. The latter is amplified via the polymerase chain reaction (PCR), and the selection process is typically repeated for 8–15 rounds. Since its first application in 1990, SELEX has undergone many novel modifications with the view to identify more specific aptamers and mak the selection process more efficient, cost-effective, and rapid. The different variants of SELEX as well as other operational and technical issues related to this procedure have been covered by various reviews [1,32,33,34,35].

Aptamers have been considered “artificial antibodies”, and considerable attention has been given to them as potential alternatives to antibodies as bioreceptors due to their low production cost, easy chemical modification, high chemical stability and binding affinity, repeatability, and reusability. The relative advantages of aptamers vs. antibodies are discussed in [1,36].

Aptamers may fold into secondary and tertiary structures with a combination of loops, stems, hairpins, pseudoknots, bumps, or G-quadruplexes, enabling the detection of the target molecule by utilizing primarily hydrogen bonding, Van der Walls interactions, and electrostatic interactions [37]. This property endows aptamers with high specificity and affinity for a variety of targets, ranging from ions to molecules of various properties, sizes, and complexities, including ions, small molecules (such as amino acids, nucleotides, antibiotics), macromolecules (peptides, proteins, nucleic acids), or whole viruses, bacteria, and cells [4,5,32,35,38,39]. Aptamers can also be readily chemically modified in order to increase the range of their targets, increase their binding affinity and conjugation efficiency to their targets or nanomaterials, and promote their stability. These strategies range from adding an active functionality at either the 3′ or 5′ terminals to several more complex chemical conjugation methods, including thiol-maleimide, carbodiimide, oxidative, thiol–gold coordination, avidin–biotin coupling, and click chemistry [1,40].

### 3.2. Aptasensors

Biosensors are bioanalytical devices containing a biological recognition moiety (such as cells, antibodies, enzymes, or oligonucleotides) that selectively reacts/binds with the target of interest. The resulting biorecognition event is converted into a measurable signal by a suitable transducer. Aptasensors, as their name suggests, utilize an aptamer as the biorecognition element. Aptasensors should be differentiated from DNA sensors or genosensors, even though aptasensors and genosensors utilize single-stranded nucleotide sequences for biorecognition. In genosensors, the target is an oligonucleotide or DNA/RNA fragment, and the biorecognition element is a complementary target oligonucleotide; the biorecognition is sequence-dependent and occurs through hybridization (formation of double-strand structure) between the bioreceptor and the target [41]. In contrast, in aptasensors, the target is an ion, molecule, or cell, and the biorecognition mechanism is structure- or conformational-dependent and not sequence-dependent [42]. Once synthesized to bind to a specific target, an aptamer can be integrated into a sensing configuration. The resulting aptasensors are used in different fields such as POC testing, food safety, and the environment [2,3,4,5].

**Figure 1:**
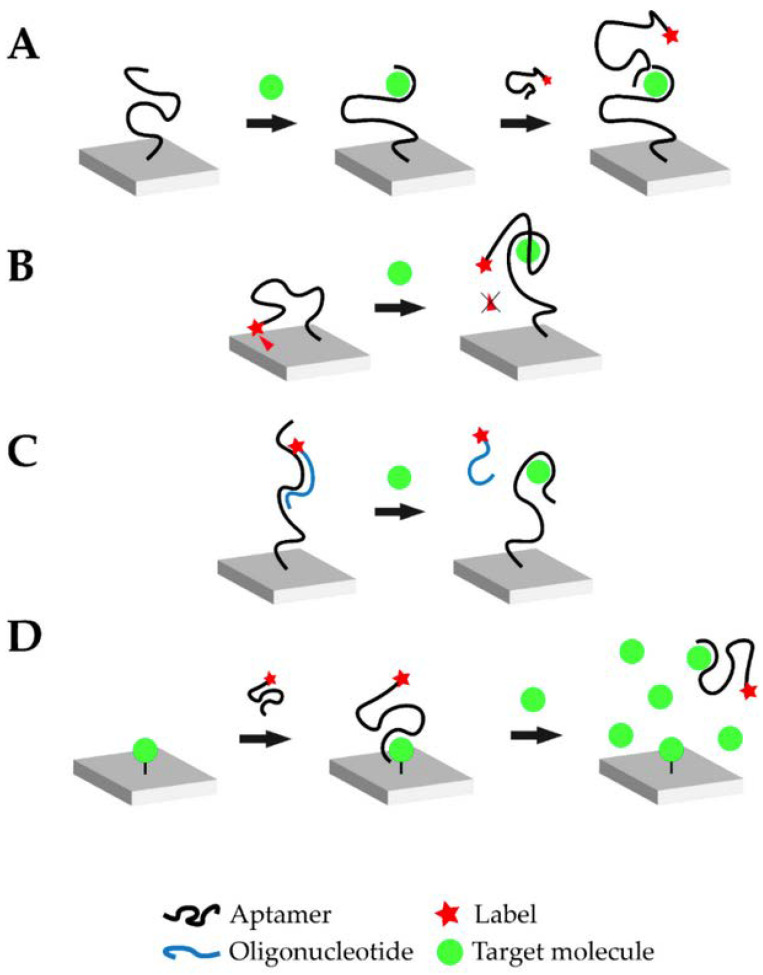
Overview of the different possible operational modes of aptasensors: (**A**) Sandwich or sandwich-like mode, (**B**) Target-induced structure switching mode, (**C**) Target-induced dissociation mode, (**D**) Competitive replacement mode (Reprinted with permission from Ref. [32]).

In aptasensors, the oligonucleic acid is commonly immobilized on the biosensor surface. Immobilization drastically improves the handling of the biosensor, and the aptamer can be regenerated more easily for multiple measurements. Once properly immobilized on the biosensor surface, the binding event between the aptamer and the target is translated into a signal via a suitable transducer. Figure 1 schematically illustrates four typical and widely used operational modes of aptasensors via which the analytical signal can be generated: sandwich or sandwich-like mode, target-induced structure switching mode, target-induced dissociation mode, and finally competitive replacement mode.

**Figure 2:**
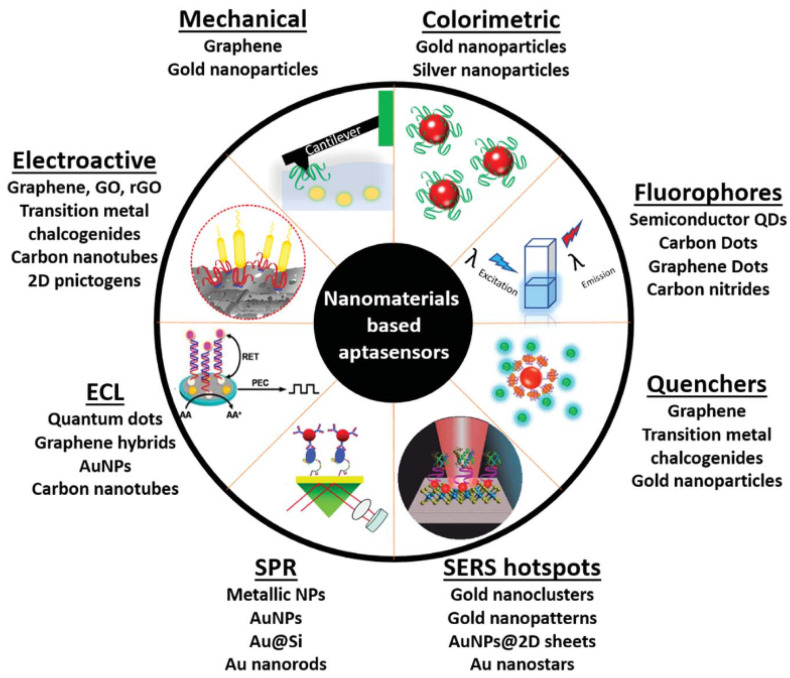
Schematic diagram of the diverse use of nanomaterials in aptasensing (Reprinted with permission from Ref. [47]).

Different strategies have been developed to enhance the operational characteristics of aptasensors [43]. The recent advances in the field of nanomaterials have also impacted the field of biosensors [44,45], as illustrated in Figure 2. These nanomaterials (such as metal and metal oxide nanoparticles (NPs), nanowires (NWs), nanorods (NRs), carbon nanotubes (CNTs), graphene oxide (GO), and quantum dots (QDs)) can contribute to a more efficient transduction process, enhance the analytical performance of sensors (sensitivity, response time, reproducibility, detection limits), and potentially lead to more miniaturized devices. Following this trend, nanomaterials have been applied as functional materials in various aptasensor platforms [33,46,47,48,49,50].

## 4. Detection Modes in Paper-Based Aptasensors

### 4.1. Optical Detection

Various detection schemes have been applied in conjunction with PADs [14], with optical detection being the most widely used [49]. Optical aptasensors provide detection of the target species based on an optical signal generated by the interaction between the aptamer and the target. Optical detection is one of the most commonly used detection approaches in aptasensing because it offers convenient coupling to various laboratory instruments such as fluorescence microscopes, CCD cameras, chemiluminometers, and photodiodes and even enables instrument-free detection with smartphones [51]. Several optical methods have been used in paper-based aptasensing, including colorimetry, fluorescence, and luminescence. Recent developments in nanomaterials have greatly facilitated and accelerated the development of optical aptasensors with enhanced operational features [33,47,48].

**Figure 3:**
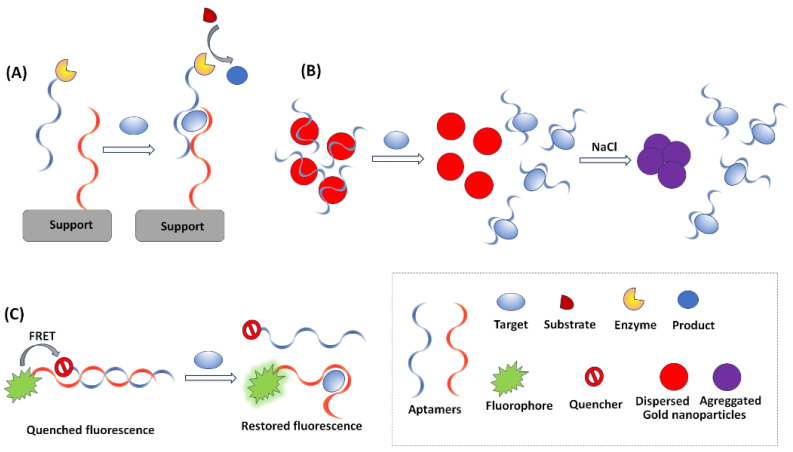
The principles of the main optical detection modes in aptasensing. (**A**) enzyme-based colorimetric aptasensor, (**B**) nanomaterial-assisted colorimetric aptasensor, (**C**) FRET-based aptasensor.

In colorimetric aptasensors, the target analyte is identified and quantified by variations in the color or the color intensity induced by the aptamer-target interaction. Colorimetric methods are widely used in aptasensing due to the simple, portable, and low-cost instrumentation required. The compatibility of colorimetric methods with low-cost reporting devices, such as smartphones and scanners, as well as with miniaturized detectors, enables the development of inexpensive and portable set-ups. Aptamers have been utilized as bioprobes in colorimetric enzyme-linked oligonucleotide assays (ELONA), also known as enzyme-linked aptamer assays (ELAA) or enzyme-linked aptamer sorbent assays (ELASA), which are the equivalent to enzyme-linked immunosorbent assays (ELISA) using antibodies [5]. ELONA relies on aptamers immobilized on a solid substrate to capture the targets, followed by binding with another enzyme-tagged aptamer (or antibody); the enzyme catalyzes the oxidation of a colorless substrate to a colored product (Figure 3A). Many colorimetric aptasensors make use of gold nanoparticles (AuNPs), whose localized surface plasmon resonance produces a bright red coloration that is highly susceptible to changes in nanoparticle size, allowing their utilization as optical probes. AuNPs are used in both the “signal-on” and the “signal change” modes. In “signal-on” aptasensors, AuNPs are attached to the target, either directly or indirectly, and cause an increase in the red color intensity [47]. The “signal change” provides a wider scope for analytical applications; it is based on the fact that AuNPs of ca. 20 nm diameter are red in color and show a blue shift with an increase in size [47]. Therefore, a distinct change in the visible spectrum could be observed along with a change in the color of the AuNPs when there is a change in the ratio of dispersed-to-aggregated AuNPs. In the absence of the target analyte, the aptamer provides a protective layer on the AuNPs, thus preventing salt-induced aggregation of the AuNPs, which remain red in color. In the presence of a target analyte, the aptamer dissociates from the AuNPs and binds with the target; therefore, the free AuNPs undergo salt-induced aggregation and their color turns blue-purple (Figure 3B).

Analytical aptasensing strategies based on fluorescence detection require more expensive instrumentation than colorimetric methods but provide excellent sensitivity. The principle of operation of such aptasensors is that some fluorophores, used as detection probes, are able to change their emission due to conformational changes of aptamers induced by binding with the target [35]. A suitable fluorophore material for aptasensor design is expected to be characterized by a high fluorescence lifetime, low photobleaching, and narrow emission bands. Although traditional organic dyes (such as Alexa Fluor 488, fluorescein, and carboxyfluorescein) can serve as fluorophores, nanomaterials (such as QDs and other semiconducting nanocrystals) are widely used because they meet the fluorophore criteria in a more satisfactory way. In particular, QDs exhibit excellent luminescence properties as a virtue of the quantum effects arising due to their tiny sizes. The fluorescence of QDs can usually be controlled by their size and shape. Due to their high fluorescence yield, QDs are widely utilized as ultra-sensitive fluorescent probes in aptasensing [47]. Some nanomaterials may be applied in the design of aptasensors by virtue of their function as quenchers in the Förster resonance energy transfer (FRET) fluorescence mode (Figure 3C). FRET is based on the coupling between a fluorophore (i.e., a visible light-emitting molecule) and a quenching molecule that absorbs visible light and emits optical energy at invisible wavelengths. A prominent material used as a quencher is GO. Single-stranded aptamers have the tendency to bind with the GO planar sheets through *π*-*π* stacking, while the planar structure of GO provides a very large surface area for molecular interactions and can harvest the radiative energy emitted by the fluorophores, quenching them in the process. The main reasons for the widespread use of GO in biosensing are its easy functionalization and conjugation, its high optical quenching ability, its excellent dispersibility in aqueous media, and its biocompatibility [47]. Several applications adopt FRET in displacement assays by exploiting the fact that the fluorophore–aptamer conjugate remains bound to GO in the quenched state in the absence of the target. The addition of target species causes the removal of the aptamer strands from GO, restoring the fluorescence signal [51].

Another optical detection technique used in conjunction with aptasensors is luminescence [52]. Two main variants of luminescence are used in paper-based aptasensors, namely chemiluminescence (CL) and electrogenerated chemiluminescence (ECL). CL sensors are based on light generated by appropriate chemical reactions between two reactants. Compared with fluorescence, CL does not require a light source and exhibits a lower background as well as higher sensitivity. ECL relies on light generated by specific electrochemical reactions; its advantages over CL include lower background signals and higher selectivity achieved by control of the driving excitation potential or current applied to the electrode.

### 4.2. Electrochemical Detection

After colorimetry, electrochemistry is the second most widely used detection method in PADs [53]. It is well known that electrochemical detection has some important advantages: it requires simple, low-cost, and portable instrumentation, offers high sensitivity and high selectivity after judicious choice of the detection technique electrode material, and can be miniaturized.

**Figure 4:**
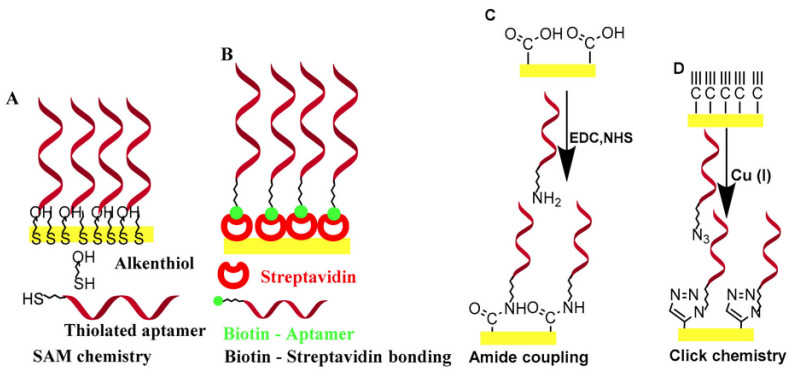
Common surface immobilization methods for aptamers. (**A**) SAM chemistry, (**B**) biotin-streptavidin bonding, (**C**) amide coupling, and (**D**) click chemistry (Reprinted with permission from Ref. [56]).

One of the most critical elements in an electrochemical aptasensor is the reporting electrode; the applicability of different types of disposable and non-disposable types of electrodes, as well as new electrode materials for aptasensing, have been reviewed [54]. A prerequisite for the development of electrochemical aptasensors is the modification of a sensing electrode surface with the aptamer biorecognition element. A wide variety of coupling chemistries can be adopted for linking aptamers to a variety of sensing electrode materials (including thiol-on-gold self-assembled monolayers of aptamers, biotin-avidin bonding, carbodiimide-mediated amine-carboxy group reaction, and click chemistry) [55,56] (Figure 4).

In terms of the electrochemical technique used, electrochemical aptasensors can be categorized as voltammetric/amperometric, impedimetric, and potentiometric [55,56,57,58]. In voltammetric/amperometric aptasensors, the aptamer-target interaction is monitored by a current generated through a redox reaction of an electrochemically active label entity. In potentiometric aptasensors, the potential difference between an indicator electrode and a reference electrode is measured and related to the target species concentration in contact with the indicator electrode. In impedimetric aptasensors, the binding event induces a change in the charge transfer resistance of the transducer/solution interface, which is monitored by electrochemical impedance spectroscopy (EIS).

A second classification relies on whether a label is used in the assay (labeled) or not (label-free) [59]. Labeled aptasensors are highly sensitive due to the signal amplification resulting from the action of the label (typically an enzyme or an electrochemically active compound). However, the modification of aptamers with tagged molecules is time-consuming and labor-intensive and may affect the binding affinity of the aptamer to the target.

**Figure 5:**
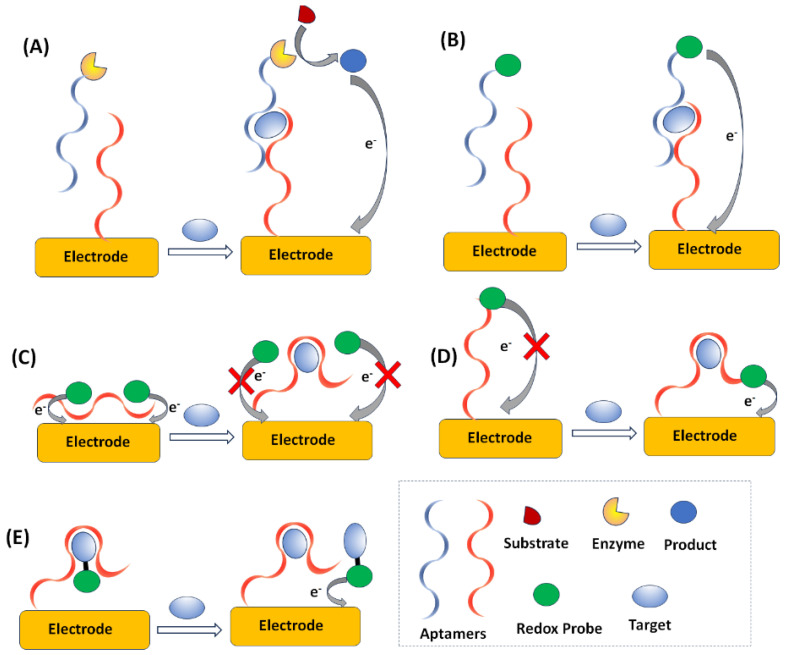
Electrochemical aptasensing modes. (**A**) formation of “sandwich” structure with electrochemical detection of an enzymatic product, (**B**) formation of “sandwich” structure with electrochemical detection of a redox probe, (**C**) aptamer folding, (**D**) electrode surface blocking, (**E**) displacement.

**Figure 6:**
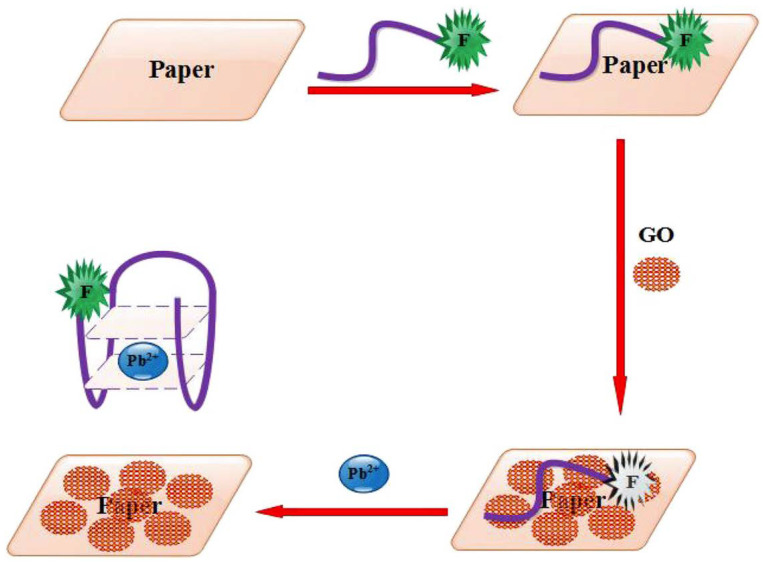
The schematic of the paper-based aptasensor combined with the FRET process for the Pb^2+^ detection (Reprinted with permission from Ref. [65]).

A final categorization is based on the aptasensing assay principles, which are exploited and can involve: the formation of “sandwich” structures; aptamer folding; electrode surface blocking; and displacement [55,56,57,58]. Sandwich assays are based on the use of a set of two probes consisting of either two different aptamers that are able to bind a target at two distinct sites or an aptamer-antibody combination. The first probe is used as a surface-immobilized capture probe. The second probe is used as a detection probe labeled with the specific redox label (or an enzyme catalyzing a reaction forming a redox-active product); if the target is present, a sandwich is formed and the redox label (or the enzymatic product) is electrochemically monitored (Figure 5A,B). Another common methodology is to exploit conformation changes in transducer-bound, redox-labeled aptamers upon the addition of a target. The conformational change induced by the binding event (e.g., folding) causes a change in the distance between the transducer, and the resulting change in the electrochemical signal is monitored (folding assays) (Figure 5C). Blocking assays are based on the aptamer-target binding causing a change in the morphology of the surface, which can be assessed electrochemically using a redox probe in solution (Figure 5D). Finally, in displacement assays, the target species in the sample compete with labeled target species for specific binding sites on the sensor surface, and the displaced species are electrochemically interrogated (Figure 6E). Signal amplification strategies exploiting the use of various nanomaterials (metal nanoparticles, QDs, magnetic nanoparticles (MNPs), carbon-based nanoparticles, and polymeric nanoparticles) have been widely reported in electrochemical aptasensing, serving as sensing platform modifiers, nanocarriers, nanocatalysts, nanotraces, magnetic accumulators, and separators [48,60].

## 5. Applications of Aptasensing PADs

### 5.1. Ions

Paper-based aptasensors have been mainly reported for the quantification of metal ions, many of them of environmental importance due to their toxicity (e.g., Pb(II), Hg(II)) or biological function (e.g., K^+^). The common methods for the determination of these cations are based on advanced spectroscopic techniques that offer low limits of detection and multi-metal determinations [61]. However, these methodologies are laboratory-based, requiring expensive and bulky equipment, trained personnel, and sample pretreatment. In contrast, aptasensing PADs can serve as sensitive, low-cost on-site diagnostic devices to acquire preliminary information on potential heavy metal contamination in many samples [38]. However, the selection of aptamer sequences for heavy metals is challenging, and in many cases the binding selectivity is low [38], while other competing low-cost and field-deployable analytical approaches are available (e.g., stripping voltammetry [62]). Table 1 summarizes the features of the reported aptasensing PADs for ions.

A paper-based calorimetric aptasensor has been fabricated for K^+^ detection using cationic dyes [63]. The cationic dye (Y5GL) serves as an aggregator, which changes the AuNP solution color from blue-purple to green. In the presence of K^+^, the aptamer dissociates from the surface of the AuNPs, so that the free AuNPs and cationic dye make the solution green. The linear range of the aptasensor was from 10 μM to 40 mM and the limit of detection (LOD) of 6.2 μM was obtained.

Another aptasensing microfluidic PAD has been reported for Pb^2+^ ions in the water [64]. It is based on the aggregation of AuNPs with NaCl, leading to a color change from red to purple in the presence of Pb^2+^ (Figure 6). Whatman No. 1 and nylon filter papers were used as the platform of this assay with a linear range from 10 nM to 1 mM for both supports; the LODs were 1.2 nm and 0.7 nm, respectively.

An aptasensing PAD, based on FRET, was proposed for the detection of Pb^2+^ [65]. The detection exploits conformational transformations of the Pb^2+^-specific aptamer that affect its binding with GO. The addition of the target Pb^2+^ induces the release of the specific aptamer from the GO surface, thus restoring the fluorescence emission. The linearity held in the ranges 5–70 pM and 0.07–20 nM with an LOD of 0.5 pM.

Finally, a CL cellulose aptasensor was introduced for Hg^2+^ detection in water using a sandwich assay made up of two aptamers [66]. A capture aptamer (S1) is immobilized on paper. When the target Hg^2+^ is captured by S1, it is tagged by the second aptamer (S2), which is modified with CL reagents (phenylene-ethynylene reagents on nanoporous silver). Finally, CL is induced via the addition of permanganate. This device allowed Hg^2+^ detection in a range of 20 nM to 0.5 μM with an LOD of 1 pM.

### 5.2. Small Molecules

Small molecules are typically organic compounds with a molecular weight of <900–1000 Daltons and comprise a large variety of natural or man-made compounds of environmental, biological, pharmaceutical, or industrial importance. Natural food contaminants (such as toxic mycotoxins, aflatoxins, and ochratoxins produced by fungi), pesticides, many pharmaceuticals (e.g., antibiotics), and human body regulators (such as vitamins and hormones) are typical examples of small molecules with high significance. The main challenges in the development of aptasensors for small molecules are related to the structure of the targets, the aptamer selection process, and the determination of the binding constant [32]. The small size of the target and the lack of functional groups available for immobilization or interaction with nucleic acids make the selection process of small molecules’ aptamers very challenging. Nevertheless, the current progress in SELEX technologies such as high-throughput sequencing (HTS) and post-SELEX optimization procedures has led to improved screening of aptamers that are selective to small molecules [67,68]. However, aptamers provide appropriate binding pockets within their tertiary structures for the recognition of small molecules and are, therefore, better small-molecule receptors compared to antibodies [43]. Typical examples of paper-based aptasensors for small molecules are gathered in Table 2.

**Table 2:** Examples of paper-based aptasensors for small molecules.

Analyte (Sample)	PAD	Type of Paper	Detection	Aptamer Sequence	Ref.
Epinephrine	NR *-wax dipping	Whatman No. 1	Colorimetric with AuNPs	32-mer (sequence NR *)	[69]
Streptomycin, tobramycin, kanamycin (milk)	Five layer-wax printing	Whatman chromatography paper No. 4, 5	Fluorescence	Str: TAGGGAATTCGTCGACGGATGCGGGG TCTGGTGTTGTGCTTTGTTCTGTCGGG TCGTCTGCAGGTCGACGCATGCGCCG Tob: GACTAGGCACTAGTC Kana: TGGGGGTTGAGGCTAAGCCGAC 78.8	[70]
Quinine, serotonin (urine, water, tomatoes, tomato juice)	Triangular-hand cutting	Glass microfiber filter paper	Paper spray-mass spectrometry	qui: 5′-GAC-AAG-GAA-AAT-CCT-TCA-ACG-AAG-TGG-GTC-3′ ser: 5′-CGA-CTG-GTA-GGC-AGA-TAG-GGG-AAG- CTG-ATT-CGA-TGC-GTG-GGT-CG-3′	[71]
Tetracycline (water) Guanosine tetra- Phosphate (cell lysate)	Circular-hole punch	Whatman No. 42	Fluorescence	tet: AUGGAAAAACAUACCAGAUUUCGAUCUGGAGAGGUGAAGAAUACGACCACCUUCCCA ppGpp: NR	[72]
Kanamycin (milk)	Strips-hand cutting	NR *	Potentiometry	5′-AGATGGGGGTTGAGGCTAAGCCGA-3′	[73]
Adenosine	Origami-wax printing and lamination	Whatman No. 1	Charge	5′-ACTCATCTGTGAAGAGAACCTGGGGGAGTATTGCGGAGGAAGGT-3′	[74]
Gentamicin	Star-shaped-hand punch	Whatman Protran	Colorimetric with AuNPs	5′-GGGACT TGGTTTAGGTAATGAGTCCC- 3′	[75]
17β-estradiol (serum)	Origami-wax printing-screen printed electrodes	Whatman No. 1	DPV	5′-SH-(CH2)6-GCTTCCAGCTTATTGAATTACACGCAGAGGTACGGCTCTGCGC ATTCAATTGCTGCGCGCTGAAGCGCGGAAGC-3′	[76]
Ochratoxin A (corn, wheat)	Circular-wax printing-screen printed electrodes	Whatman No. 1	DPV	5′-SH-(CH2)6-GATCGGGTGTGGGTGGCGTAAAGGGAGCATCGGACA-3′	[77]
Adenosine triphosphate	Origami/wax printing/screen-printed electrodes	Whatman chromatography paper No. 1	ECL	capture: 5′-HS-(CH_2_)_6_-ACCTGGGGGAGTAT-3′; probe: 5′-TGCGGAGGAAGGT-NH_2_-3′	[78]
Ochratoxin A (corn, groundnut)	Microfluidic-photoresist	Whatman filter paper	Colorimetric with AuNPs	5′-GATCGGGTGTGGGTGGCGTAAAGGGAGCATCGGACA-3′	[79]

* not reported.

Saraf et al. proposed a simple label-free colorimetric aptasensor for epinephrine detection [69]. The detection of epinephrine is based on the interaction with aptamer-functionalized AuNPs. A change in color from red to blue was observed in the solution with increasing concentrations of epinephrine, and the LOD was 0.9 nM.

In another work, a fluorescent paper-based aptasensing method was developed for simultaneous aminoglycoside detection (streptomycin, tobramycin, and kanamycin) [70]. The paper platform consists of five paper layers and four parallel channels. Aptamer/graphitic carbon nitride nanosheet-modified layers can catalyze o-phenylenediamine to fluorescent 2,3-diaminophenazine (DAP) in the presence of H_2_O_2_. The peroxidase-like activity is reduced when the aptamer is detached from the nanosheets as a result of its binding with the target molecules. The calibration curves for streptomycin, tobramycin, and kanamycin were linear in the ranges 0.01–30 ng mL^−1^, 0.1–150 ng mL^−1^, and 0.05–150 ng mL^−1^, respectively, and the LODs were estimated as 0.023, 0.069, and 0.045 ng mL^−1^, respectively.

**Figure 7:**
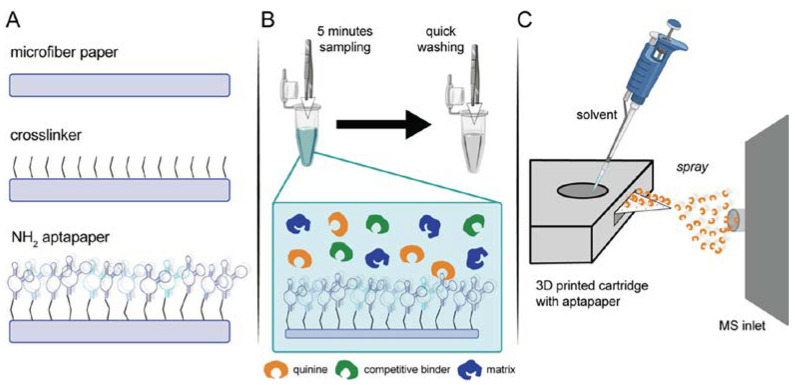
Aptapaper methodology. (**A**) General outline of aptapaper synthesis, p-phenylene di-isothiocyanate is used as a cross-linker between the paper and the amino-modified aptamer; (**B**) Target binding with aptapaper. Five minutes of dipping in the solution followed by a washing step to get rid of unspecific binders; (**C**) Paper spray analysis of aptapaper. Acidified solvent and the applied high voltage were used to release the target from the aptamer) (Reprinted with permission from Ref. [71]).

Martínez-Jarquín et al. introduced a paper platform (called “aptapaper”) modified with aptamers for the separation, preconcentration, and semi-quantitation of quinine and serotonin [71] (Figure 7). After preconcentration of the targets on the aptapaper, they were detected by paper spray ionization coupled with high-resolution mass spectrometry. The LODs were 81 pg mL^−1^ and 1.8 ng mL^−1^ for quinine and serotonin, respectively.

A portable paper-based sensor system has been reported for the rapid detection of tetracycline and guanosine tetraphosphate [72]. The target detection is performed on RNA-modified filter papers using a target-binding aptamer with fluorogenic RNA. The binding of the target with the aptamer induced the folding of the RNA, which activated the fluorescence of a fluorophore (DFHBI-1T). This sensor was used for the selective determination of tetracycline with a linear range of 0.1–0.8 mM and of guanosine tetraphosphate in the range of 0.1–10 μM.

**Figure 8:**
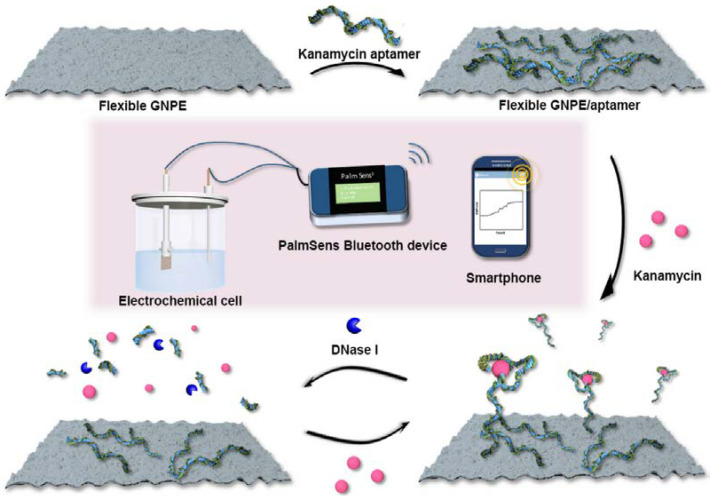
Schematic illustration of the principle of flexible freestanding graphene paper-based potentiometric enzymatic aptasensor for nuclease-based amplification detection of kanamycin (Reprinted with permission from Ref. [73]).

In addition, a potentiometric aptasensor using a graphene paper support was proposed to detect kanamycin [73]. A nuclease-assisted amplification process was implemented in order to significantly improve the detection sensitivity via the catalytic recycling reaction of the target induced by the nuclease (DNase I) (Figure 8). This aptasensor exhibits linear ranges in the 0.03–20 pg mL^−1^ and 20–150 pg mL^−1^ intervals with an LOD of 30.0 fg mL^−1^.

A self-powered origami PAD was implemented by Liu et al. to determine adenosine [74]. The device uses an aptamer to recognize an analyte, GOx to modify the relative concentrations of the [Fe(CN)_6_]^3−^/[Fe(CN)_6_]^4−^ redox couple, and a digital multimeter to record the result of the assay. This device offers an LOD for adenosine of 11.8 μM.

A flower-shaped microfluidic paper biosensor for gentamicin in milk [75] was developed employing colorimetric detection of the salt-induced aggregation of AuNPs with an LOD of 300 nM.

Ming et al. described a folding, label-free electrochemical aptasensor to detect estradiol [76]. Amine-functionalized single-walled carbon nanotube/methylene blue/AuNPs were immobilized on the working electrode to increase its detection sensitivity and immobilize the aptamer. The principle is based on a decrease in the voltametric response as soon as the aptamer and target combine. A linear range of 0.01–500 ng mL^−1^ and an LOD of 5 pg mL^−1^ were achieved.

**Figure 9:**
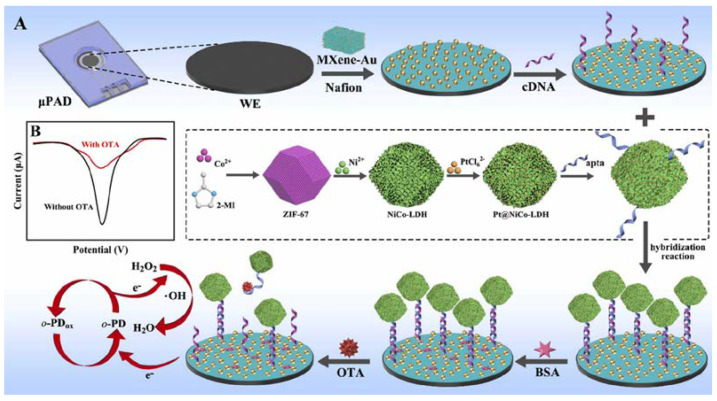
Schematic illustration of the process for the construction of the electrochemical paper-based aptasensor (**A**), and electrochemical “signal on/off” detection principle (**B**) (Reprinted with permission from Ref. [77]).

Zhang et al. have fabricated an electrochemical aptasensor to detect ochratoxin A [77] (Figure 9). The complementary aptamer was attached to a MXene-Au electrode decorated with Pt nanoparticles (NPs) anchored in hollow structures of NiCo-layered double hydroxides as signal amplification materials via Au-S bonds. Then, aptamer (apta)-binding Pt@NiCo-LDH with peroxidase-like activity was immobilized through hybridization to trigger the “signal on” state, generating a significant electrochemical signal. The presence of ochratoxin A enabled the dissociation of the aptamer-complimentary aptamer hybrid, releasing signal amplification labels to achieve a “signal off” state. The aptasensor exhibited a linear range from 20 fg mL^−1^ to 100 ng mL^−1^ and an LOD of 8.9 fg mL^−1^.

An origami ECL aptasensor was developed for adenosine triphosphate using an AuNPs-modified paper-working electrode [78]. The sandwich assay employs a thiolated capture aptamer that is immobilized on the working electrode and an amino-modified probe aptamer with ECL Pt–Ag alloy nanoparticle labels. The presence of the target induces hybridization of the two fragments, leading to enhancement of the ECL intensity. The LOD was 0.1 pmol L^−1^, and the linear range was from 0.5 pmol L^−1^ to 7.0 nmol L^−1^.

Finally, an aptamer was used as a molecular recognition element coupled with the target-induced color change of AuNPs for colorimetric detection of ochratoxin A in a microfluidic paper-based analytical device [79]. Although the device can only provide a Yes/No qualitative result, it has the potential to rapidly detect ochratoxin A without pre-treatment steps.

### 5.3. Large Molecules and Proteins

The field of protein detection on PADs is dominated by immunosensors, which utilize antibodies as the biorecognition element [80]. Antibodies are considered the “gold standard” since their high affinity and specificity to bind their target analyte have naturally evolved over long periods of time, and, in addition, there has been accumulated expertise on their use for biosensor design for many decades [50]. Recent studies comparing the relative performance of aptamers and antibodies in biosensor design (in terms of selectivity and sensitivity) are inconclusive because the results depend strongly on many experimental variables, the most important being the proper integration of the biosensing element with the transducer [36]. Table 3 summarizes some examples of paper-based aptasensors for large molecules and proteins.

A syringe-based colorimetric paper aptasensor has been reported for the assay of the malaria biomarker *Plasmodium falciparum* lactate dehydrogenase [81]. The target protein is captured by the aptamer immobilized on microbeads and is detected by a color change due to its enzymatic activity upon a development reagent. The device responds to concentrations of the biomarker spanning four orders of magnitude and achieves an LOD of 5 ng mL^−1^.

In another interesting work, aptamers were selected to discriminate *B. caeruleus* (common krait) venom from cobra, Russell’s, and saw-scaled viper’s venom [82]. The selected aptamers (against the β-bungarotoxin present in the specific venom) were used as a molecular recognition element in a colorimetric paper-based devicethat was able to detect 2 ng krait venom.

**Table 3:** Examples of paper-based aptasensors for large molecules and proteins.

Analyte (Sample)	PAD	Type of Paper	Detection	Aptamer Sequence	Ref.
*Plasmodium* *falciparum* lactate dehydrogenase (blood)	circular-hole punch	Whatman 3MM chromatography paper	colorimetric of enzymatic activity	5′Biotin- CTG GGC GGTAGAACCATAGTGACCCAGCCG TCTAC-3′	[81]
β-bungarotoxin (venom)	circular-wax printing	Whatman filter paper No. 4	colorimetric assay with streptavidin-HRP- TMB	5′-CATACAAACGGAAATTCCGATTTAGTCTTTATGATCTTGATGC-3′ 5′-GGACAGAAAAAAAAAAAGACAAAGAAGAGAGAGGGAGATGGGGCTCAT-3′	[82]
Osteopontin (serum)	circular-hand cutting	Fanoia S300 paper	colorimetric with Bradford reagent	5′-Thiol-AAAAAAAAAA TGT GTG CGG CAC TCC AGT CTG TTA CGC CGC-3′	[83]
platelet-derived growth factor (serum)	circular-wax printing	nitrocellulose HF 180	colorimetric- horseradish peroxidase-mimicking DNAzyme- H_2_O_2_- hemin	5′-ATATA GTAGA AACCA CTATC GACTC AGGCT ACGGC ACGTA GAGCA TCACC ATGAT CCTGT AGTATCAATC CTTCG CCGTC-3′ 5′-ATATA GTAGA AACCA CTATC GACTC AGGCT ACGGC ACGTA GAGCA TCACC ATGAT CCTGT AAACCCAACC CGCCC TACCC TAAA-3′	[84]
arachin, β-lactoglobulin, tropomyosin (NR)	origami-wax printing	Whatman chromatography paper	colorimetric with AuNPs	arachin: TCG CAC ATT CCG CTT CTA CCG GGG GGG TCG AGC GAG TGA GCG AAT CTG TGG GTG GGC CGT AAG TCC GTG TGT GCG AA β-lactoglobulin: ATA CCA GCT TAT TCA ATT CGA CGATCG GAC CGC AGT ACC CAC CCA CCA GCC CCA ACA TCA TGC CCA TCC GTG TGT GAG ATA GTA AGT GCA ATC T Tropomyosin: TAC TAA CGG TAC AAG CTA CCA GGCCGC CAA CGT TGA CCT AGA AGC ACT GCC AGA CCC GAA CGT TGA CCT AGA AGC	[85]
*Plasmodium* lactate dehydrogenase (NR)	rectangular-hand cutting	Printer paper	FRET	5′- GTT CGA TTG GAT TGT GCC GGA AGT GCT GGCTCG AAC—FAM—3′	[86]
mucin-1 (serum)	origami-wax printing-screen-printed electrodes	Whatman chromatography paper No. 1	ECL	5′-GCAGTTGATCCTTTGGATACCCTGG-3′	[87]
carcinoembryonic antigen (serum)	rectangular--wax printng-screen-printed electrodes	Whatman chromatography paper No. 1	ΕCL	5′HS-(CH2)6-ATA CCA GCT TAT TCAATT-3′ 5′HS-(CH2)6-CCC ATA GGG AAG TGG GGG A-3′	[88]
immunoglobulin E (serum)	circular/marker plotting	Whatman chromatography paper No. 1	FRET	5′-NH_2_-AAAAAGGGGCACGTTTATCCGTCCCTCCTAGTGGCGTGCCCC-3′	[89]
hemoglobin A1 (blood)	NR *	graphite paper	DPV with Fe(CN)_6_^3−^/Fe(CN)_6_^4−^ probe	5′-SH-TGGCAGGAAGACAAA- CACATCGTCGCGGCCTTAGGAGGGGCG- GACGGGGGGGGGCGTTGGTCTGTGGTGCTGT-3′	[90]
prostate specific antigen (serum)	microfluidic/wax printing/screen-printed electrodes	Whatman chromatography paper No. 1	DPV	5′-ATT AAA GCT CGC CAT CAA ATA GC-3′	[91]
carcinoembryonic antigen (serum)	circular/laser cutting	Whatman chromatography paper	DPV	5′-AGATACCAGCTTATTCAATTCCGCTGCTGGTATCT-3′	[92]
carcinoembryonic antigen (serum)	circular/as received	Whatman qualitative filter paper No. 3	EIS	5′-NH_2_-GAC GAT AGC GGT GAC GGC ACA GAC GTC CCG CAT CCT CCG-3′	[93]
Thrombin (serum)	origami/wax printing/screen-printed electrodes	Whatman chromatography paper No. 2	PEC	5′-GGT TGG TGT GGT TGG AGA AGA AGG CCA ACC ACA CCA ACC GAT CC-3′	[94]
prostate specific antigen (serum)	origami/wax printing/screen-printed electrodes	chromatographic paper	PEC	5′-SH-TTAATTAAAGCTCGCCATCAAATAGC-3′	[95]
vascular endothelial growth factor 165 (mesenchymal stem cells culture)	circular and microfluidic/wax printing	Whatman filter paper No. 1	fluorescence	5′-TGTGGGGGTGGACTGGGTGGGTACCGTCACTCGCCTCGCACCGTCC- Biotin—3′	[96]
epidermal growth factor receptor (serum)	origami/wax printing/screen-printed electrodes	Whatman chromatography paper No. 1	DPV	5′-TAC CAG TGC GAT GCT CAG TGC CGT TTCTTC TCT TTC GCT TTT TTT GCT TTT GAG CAT GCT GAC GCA TTC GGT TGA C-3′	[97]

* not reported.

A novel cellulose-based aptasensor for the colorimetric detection of the cancer biomarker protein osteopontin has been reported [83]. The paper was chemically modified with (mercaptopropyl)methyldimetoxisilane to attach the thiolated aptamer. Colorimetric detection was performed using a Bradford reagent. The linear range was 5–1000 ng mL^−1^ and the LOD was <5 ng mL^−1^.

In addition, Li et al. have reported on a colorimetric aptamer-based assay for the detection of platelet-derived growth factor, a target protein on bioactive paper [84]. The aptamer was self-assembled onto graphene oxide, followed by desorption induced by the specific binding of the target. The released aptamer hybridizes to paper-bound DNA primers, thus initiating a rolling circle amplification reaction to produce a long DNA molecule containing multiple horseradish peroxidase-mimicking DNAzyme moieties that catalyze the oxidation of substrates by H_2_O_2_ in the presence of hemin. This device can achieve detection of the target protein at 100 pmol L^−1^ with a linear range from 0.001 to 10 nmol L^−1^.

A microfluidic paper-assisted analytical device was developed to determine the food allergens arachin, β-lactoglobulin, and tropomyosin using a colorimetric assay [85]. AuNPs were conjugated with biotinylated aptamers and incubated with the sample. In the absence of analytes, the AuNP-aptamer conjugates will not adsorb on GO, but in the presence of analytes, the AuNP-aptamer conjugates will adsorb on GO-forming aggregates. Allergens were determined in the 25–1000 nmol L^−1^ range with LODs ranging from 6.2 to 12.4 nmol L^−1^.

A paper-based aptasensor with FRET detection has been proposed for the detection of the malaria biomarker *Plasmodium* lactate dehydrogenase [86]. Fluorescently labeled aptamers towards the target protein were incubated with fluorescence-quenching MoS_2_ nanosheets that reduced the fluorescence of the aptamers. If the sample contains the target, this will bind to the aptamers and restore the fluorescence.

**Figure 10:**
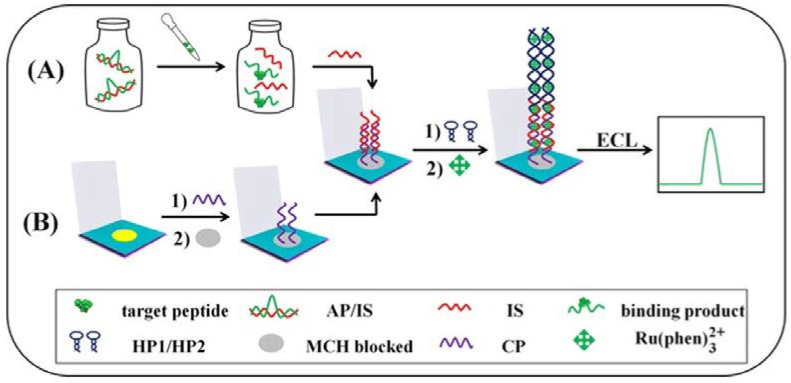
Schematic representation of the modified μ-PADs for detecting peptides: (**A**) after the specific recognition between the target mucin-1 and AP/IS, the free IS is taken into the modification processes, and finally the ECL signals are gained; (**B**) the detailed electrode modification steps (Reprinted with permission from Ref. [87]).

Ma et al. have developed an electrochemiluminescence (ECL) aptasensor for the peptide mucin-1, which is an important cancer marker [87] (Figure 10). The detection is based on the release of a strand from a target-specific aptamer in the presence of the peptide. This strand triggers a hybridization chain reaction between two hairpin probes, which is detected via an ECL probe (Ru(phen)_3_^2+^). The device enables the detection of mucin-1 in the range of 25–50 nM with an LOD of 8.33 pM.

Another paper-based bipolar ECL aptasensor for carcinoembryonic antigen has been reported [88]. An aptamer immobilized on paper is used to capture the antigen. A conjugate of a second aptamer with gold-coated Fe_3_O_4_ nanoparticles is captured by the antigen and attached to the cathode of a bipolar cell, catalyzing the reduction of H_2_O_2_ and enhancing the ECL emission at the anode. This aptasensor allows detection in a range of 0.1–15 ng mL^−1^ with an LOD of 0.03 pg mL^−1^.

A FRET protocol was adopted to develop a paper-based aptasensor for immunoglobulin E [89]. Luminescent upconversion nanoparticles serve as energy donors and carbon nanoparticles as energy acceptors to quench the fluorescence. Upon exposure to immunoglobulin E, the luminescence is recovered. The aptasensor allows the determination of the target in the range of 0.5–80 ng mL^−1^.

**Figure 11:**
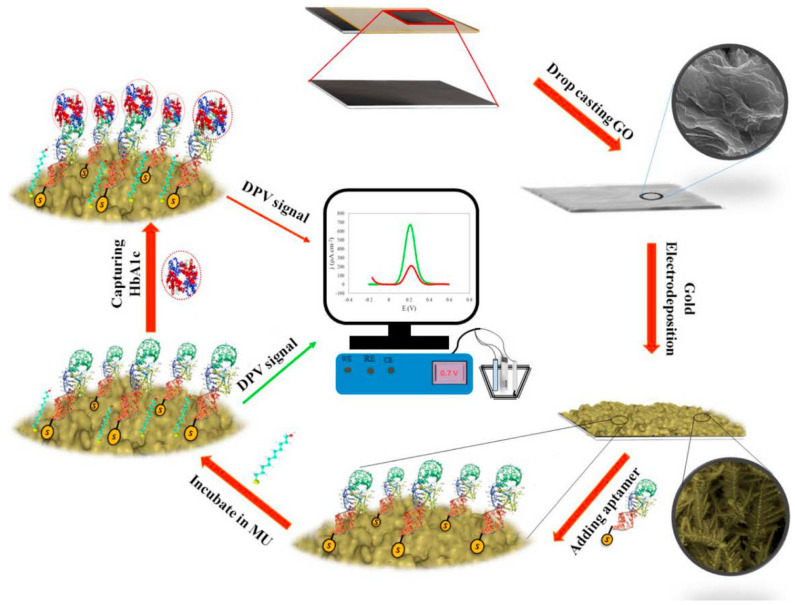
A schematic representation of the main steps for modification of GS to fabricate the hemoglobin A1c nanobiosensor. First, GO was cast coated on the graphite sheet electrode (GS) and then, gold nanostructure was electrodeposited over GS/GO. Next, thiolated DNA aptamer as bioreceptor was added to the modified electrode. The electrode was then incubated in 11-mercapto-1-undecanol (MU) solution to prevent non-specific binding. In the next step, HbA1c was added to be captured with the aptamer. Finally, the capturing of HbA1c was sensed using DPV technique (Reprinted with permission from Ref. [90]).

An electrochemical aptasensor was proposed to detect hemoglobin A1c [90] (Figure 11). A nanocomposite of reduced graphene oxide and gold was electrochemically deposited on graphite paper and used to immobilize an aptamer. In the presence of the target the DPV current of the Fe(CN)_6_^3−^/Fe(CN)_6_^4−^ probe was reduced. The linear range was 1 nmol L^−1^–13.83 μmol L^−1^ and the LOD was 1 nmol L^−1^.

**Figure 12:**
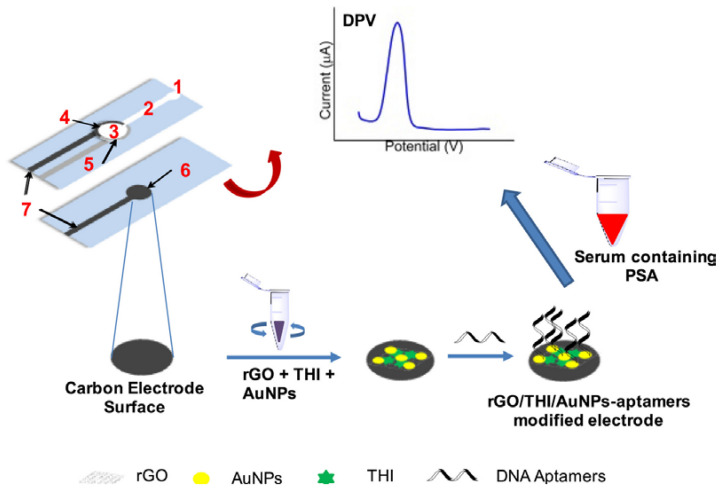
Fabrication and modification process of the microfluidic paper-based aptasensor and the typical response of the detection of analyte (1) injection port; (2) microfluidic channel; (3) reaction site; (4) screen-printed carbon counter electrode; (5) screen-printed reference electrodes; (6) working electrode; (7) screen-printed electrode-lead (Reprinted with permission from Ref. [91]).

Wei et al. have proposed a microfluidic electrochemical aptasensor for prostate specific antigen (PSA) detection [91] (Figure 12). AuNPs/reduced graphene oxide (rGO)/thionine composites were coated onto the working electrode for the immobilization of the aptamer probe. Thionine serves as an electrochemical mediator of the recognition event between the aptamer and the target. The LOD was 10 pg mL^−1^, and the linear range was 0.05 to 200 ng mL^−1^.

An electrochemical sensing platform has been reported that can detect carcinoembryonic antigen and other biomarkers [92]. A hairpin probe (containing the specific aptamer sequence) binds to the target and the binding event induces a conformational change of the probe exposing its occluded stem region. The exposed domain triggered a polymerization reaction generating DNAzyme strands that produce an amplified signal response.

A paper-based electrochemical aptasensor for carcinoembryonic antigen has been fabricated [93]. A graphene/poly (3,4-ethylenedioxythiophene):poly(styrenesulfonate) (PEDOT:PSS)-modified paper serves as the conductive substrate for the aptamer immobilization. The target binding is followed by EIS with a linear range 0.77–14 ng mL^−1^ and an LOD of 1.06 ng mL^−1^ in serum.

A photoelectrochemical cellulose-based aptasensing platform was proposed to detect thrombin by Xue et al. using a dual electron-transfer tunneling distance regulation (ETTDR) and aptamer target-triggering nicking enzyme signaling amplification (NESA) strategy [94]. In the presence of TB, a large number of secondary tDNA pieces are generated and hybridized with CeO_2_-labeled hairpin DNA immobilized on the electrode surface causing an amplified photocurrent to decrease. The aptasensor has linear range of 0.02–100 p mol L^−1^ with an LOD of 6.7 fmol L^−1^.

Another photoelectrochemical aptasensor was proposed for prostate-specific antigen based on TiO_2_/black phosphorus quantum dots (TiO_2_–BPQDs) was proposed for PSA detection [95]. Carbon nanotubes, TiO_2_, black phosphorus quantum dots, and capture DNA were sequentially immobilized on paper to bind AuNPs-modified aptamer. Upon adding the target, the aptamer dissociates from the electrode leading to photocurrent amplification. The linear range is 0.005–50 ng mL^−1^ and the LOD is 1 pg mL^−1^.

Azuaje-Hualde et al. have developed a cellulose microfluidic paper-based analytical device for the detection of Vascular Endothelial Growth Factor (VEGF) [96]. A three-part aptamer structure was designed with an aptameric sequence specific for the target which hybridizes with a fluorescent strand and a quencher strand. The presence of the target triggers the displacement of a quencher strand and increase in the fluorescence intensity. The LOD was 137 ng mL^−1^ and the linear range was 0.1–5 mg mL^−1^.

An origami electrochemical paper-based aptasensor was fabricated for label-free detection of epidermal growth factor receptors [97]. Amino-functionalized graphene/thionine/AuNPs nanocomposites were used to modify the working electrode and facilitate the immobilization of specific thiol-modified aptamers. The principle of detection was the inhibition of the electron transfer rate with the formation of the aptamer–antigen bioconjugates. The biosensors enabled detection at 5 pg mL^−1^ and a linear range of 0.05–200 ng mL^−1^.

### 5.4. Cells and Bacteria

The targets of aptamers for the detection of cells can be specific proteins or receptors at the surface of the cells, bacterial virulence factors or even the very cells themselves, and the SELEX selection process should be tailored according to the target selection [5]. A selection of applications using PAD aptasensors for cells and bacteria is provided in Table 4.

**Table 4:** Examples of paper-based aptasensors for cells and viruses.

Analyte (Sample)	PAD	Type of Paper	Detection	Aptamer Sequence	Ref.
human breast adenocarcinoma cells (MCF-7) (serum)	origami/wax printing/screen-printed electrodes	Whatman chromatography paper No. 114	ECL	5′-GCA GTT GAT CCT TTG GAT ACC CTG GTT TTT TTT TTT-HS-3′	[98]
human breast adenocarcinoma cells (MCF-7) (blood)	origami/wax printing/screen-printed electrodes	Whatman chromatography paper No. 114	ECL	5′- GCA GTT GAT CCT TTG GAT ACC CTG GTT TTT TTT TTT -HS-3′	[99]
human acute promyelocytic leukemia cells (HL 60) (NR)	origami/wax printing/screen-printed electrodes	Whatman chromatography paper No. 114	DPV	5′-ATCCAGAGTGACGCAGCATGCCCTAGTTACTACTACTCTTTTTAGCAAACGCCCTCGCTTTGGACACGGTGGCTTAGT-3′	[100]
*Listeria monocytogenes* (milk, cheese)	rectangular/wax printing/screen-printed electrodes	GSM 210 paper	EIS	5′-NH_2_-ATC CAT GGG GCG GAGATG AGG GGG AGG AGG GCG GGT ACC CGG TTGAT-3′	[101]
Zika virus (NR)	Rectangular/hand-cutting	printer paper	potentiometric	32-mehr	[102]
human breast adenocarcinoma cells (MCF-7) (serum)	Origami/wax printing/screen-printed electrodes	Whatman chromatography paper No2	DPV, colorimetric	5′-SH-CACTACAGAGGTTGCGTCTGTCCCACGTTG TCATGGGGGGTTGGCCTG-3′ 5′-biotin-TTTTTTGCAGTTGATCCTTTGGATACCCTGGTTTGCAAAGCTTACGGCATACGT-3′	[103]
MCF-7 cells, K562 cells (blood)	multi-layered/wax printing/screen-printed electrodes	NR *	DPV, colorimetric	MCF-7: 5′-NH_2_-C_6_H1_2_-CAC TAC AGA GGT TGC GTC CCA CGT TGT CCC ACG TTG TCA TGG GGG GTT GGC CTG-3′ K562: 5′-NH_2_-TTT TTT TTT TAC AGC AGA TCA GTC TAT CTT CTC CTG ATG GGT TCC TAT TTA TAG GTG AAG CTGT GGC-3′ TGG CTG GGG GGC GTT	[104]

* not reported.

Two similar paper-based ECL aptasensors were developed to detect cancer cells [98,99]. Aptamers are immobilized on the AuNPs-modified electrode via Au-S bonds and capture the target cells. Concanavalin A-labeled AuPd alloy nanoparticles bind to the captured cells amplifying the ECL signal. The devices can perform detection in the range of ~450–1.0 × 10^7^ cells mL^−1^ with an LOD of ~250 cells mL^−1^.

The same group has reported a paper-based voltammetric sandwich assay to detect human acute promyelocytic leukemia cells [100]. The Au-paper electrode is modified with aptamers to capture the cancer cells and horseradish peroxidase-labeled folic acid binds to the captured cells (via recognition of folic by folate receptors on the cell surface) which catalyzes the oxidation of o-phenylenediamine by H_2_O_2_; the enzymatic product is monitored by differential pulse voltammetry. The device enables detection in a range of 5.0 × 10^2^–7.5 × 10^7^ cells mL^−1^ with an LOD of 350 cells mL^−1^.

**Figure 13:**
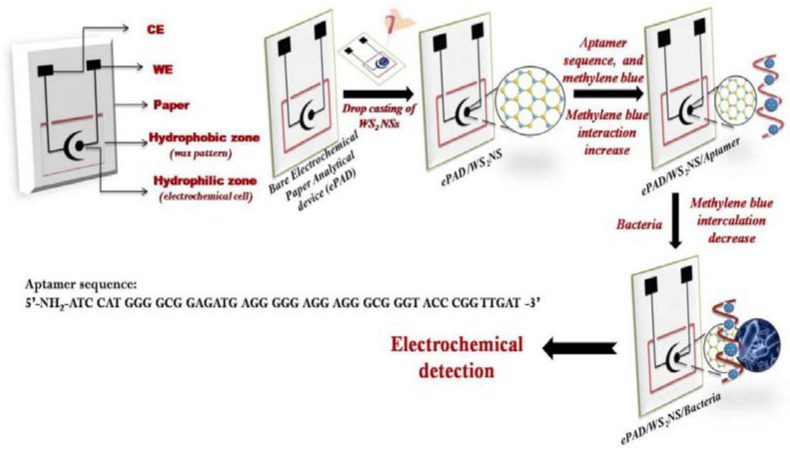
Schematic representation of the fabrication of the screen-printed paper-based aptasensor for the detection of Listeria monocytogenes (Reprinted with permission from Ref. [101]).

A novel aptasensor based on an electrochemical paper-based analytical device has been proposed for the detection of *Listeria monocytogenes* [101] (Figure 13). The paper substrate was modified with a tungsten disulfide/aptamer hybrid and detection was performed with EIS using methylene blue as a probe. A LOD of 4.5 CFU mL^−1^ and a range of 10–10^8^ CFU mL^−1^ were obtained.

A paper-based potentiometric sensor was fabricated to detect the Zika virus with an LOD of 2.4 × 10^7^ [102]. The sensor consists of 2 segments of paper (sample and reference segments) with conducting silver paint contact patches on two ends and impregnated with aptamers against Zika. When the virus is added to the sample region, a Nernstian potential difference is generated between the sample and reference regions.

**Figure 14:**
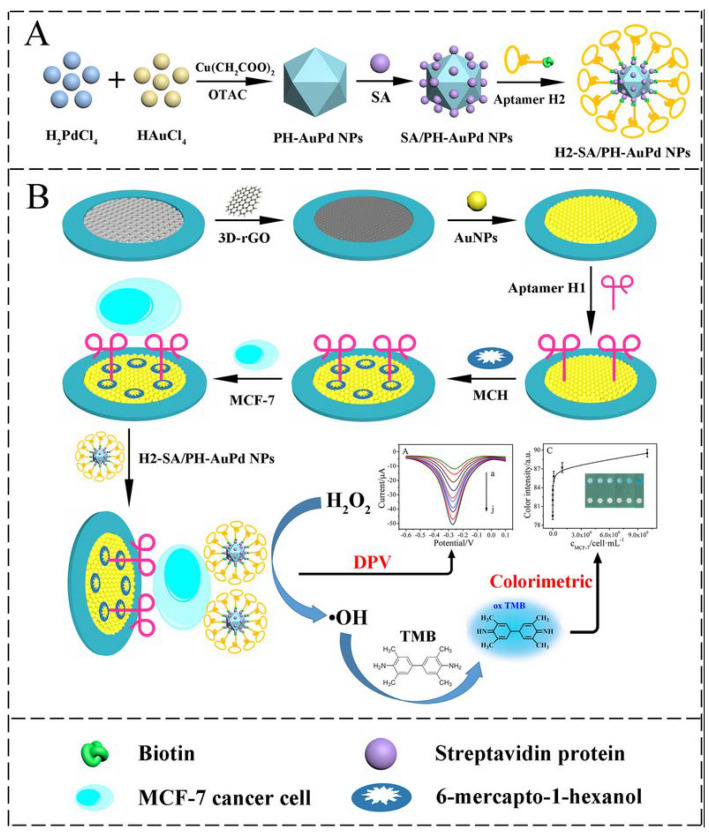
Schematic illustration of the fabrication of the dual-mode cytosensor: (**A**) preparation procedure of H1−SA/PH−AuPd NPs; (**B**) the detection principle for MCF−7 cells and the strategy of signal detection (Reprinted with permission from Ref. [103]).

Wang et al. have reported on a paper-based dual-mode cyto-aptasensor for simultaneous electrochemical and colorimetric detection of breast cancer MCF-7 cells [103] (Figure 14). The on-paper working electrode is modified with reduced graphene oxide (rGO) and AuNPs to bind a first aptamer and capture the target cells. AuPd alloy nanoparticle detection probes combine with a second aptamer and are immobilized on the captured MCF-7 cells. The probes catalyze the oxidation of H_2_O_2_ resulting in an amplified electrochemical signal. In addition, the generated •OH can also produce a colorimetric signal. This device enables a linear detection range of 50–10^7^ cells mL^−1^ with an LOD of 20 cells mL^−1^.

Another dual-mode aptasensor was developed for MCF-7 and K562 cells by Li et al. [104]. The paper-based device was fabricated in six layers. For electrochemical detection, an Au-modified working electrode was used to bind a target-specific aptamer. The complementary DNA strand, labeled with methylene blue (MB), is hybridized on the electrode and is released in the presence of target cells. Aptamer-labeled Pd–Pt nanoparticles that are loaded with ferrocene (Fc) are linked onto electrode, resulting in an increased Fc/MB current intensity ratio. For colorimetric detection, H_2_O_2_ is added to the paper and, in the presence of the target cells, more aptamer-labeled Pd–Pt nanoparticles are linked to the electrode, resulting in increased consumption of H_2_O_2_ and decreased consumption of the sealing reagent (AgNPs). The cell concentration can be calculated based on the distance that the liquid moves. This aptasensor enables detection of MCF-7 and K562 cells in ranges of 150–1.0 × 10^7^ and 220–7.0 × 10^6^ cells mL^−1^ with LODs of 117 and 140 cells mL^−1^, respectively.

### 5.5. Multiplexed Assays

Multiplexed assays for the simultaneous detection of more than one analyte are extremely important. A prominent example is clinical analysis, in which several biomarkers are often necessary to monitor specific diseases (such as cancer, cardiovascular disorders, or diabetes). Another typical example is food safety since it is desirable to monitor many toxic compounds that can potentially co-exist in foodstuffs (such as mycotoxins, antibiotics, and bacteria). Apart from their higher diagnostic potential, such multi-analyte assays provide lower cost per test and higher throughput than single-analyte assays. Different multiplexed aptasensing strategies have been reported so far [105]. Table 5 summarizes applications of multiplexing cellulose-based aptasensors.

**Table 5:** Examples of paper-based multiplex aptasensors.

Analyte (Sample)	PAD	Type of Paper	Detection	Aptamer Sequence	Ref.
Hg^2+^, Ag^+^ (human serum, water, milk)	square/craft punch	Whatman No 1	FRET/GO	Hg^2+^: 5′-FAM-TTT TTT TTT TTT-3′ Ag^+^: 5′-FAM-CCC CCC CCC CCC-3′	[106]
lysozyme, ß-conglutin lupine, okadaic acid, brevetoxin (egg white, mussels, sausages, bread)	rectangular/hand-cutting	Whatman chromatography paper	FRET	lys: 5′-AGC AGC ACA GAG GTC AGA TG GCA GCTAAG CAG GCG GCT CAC AAA ACC ATT CGCATG CGG C CCT ATG CGT GCT ACC GTG AA-3′ ß-congl: 5′-AGC TGA CAC AGC AGG TTG GTG GGG GTGGCT TCC AGT TGG GTT GAC AAT ACG TAG GGA CAC GAA GTC CAA CCA CGA GTC GAG CAA TCT CGA AAT-3′ okadaic acid: 5′-CAG CTC AGA AGC TTG ATC CTA TTT GACCAT GTC GAG GGA GAC GCG CAG TCG CTACCA CCT GAC TCG AAG TCG TGC ATC TG-3′ brevet: 5′-ATA CCA GCT TAT TCA ATT GGC CAC CAAACC ACA CCG TCG CAA CCG CGA GAA CCG AAG TAG TGA TCA TGT CCC TGC GTG AGA TAG TAA GTG CAA TCT-3′	[107]
carcinoembryonic antigen, neuron-specific enolase (serum)	multi-layer microfluidic/wax printing/screen-printed electrodes	Whatman chromatography paper No1	DPV	CEA: 5′-ATA CCA GCT TAT TCA ATT-3′, NSE: 5′-CGG TAA TAC GGT TAT CCA CAG AAT CAG GGG-3′	[108]
four glycans on K562 cell surface (cell culture)	origami/wax printing/screen-printed electrodes	Whatman chromatography paper No114	DPV	5′-HS-TTT TTT TTT TAC AGC AGA TCA GTC TAT CTT CTC CTG ATG GGT TCC TAT TTA TAG GTG AAG CTG T-3′	[109]
*E. coli* O157:H7, *S. Typhimurium* (NR)	star-shaped/wax printing	Whatman filter paper No1	colorimetric with AuNPs	*E. coli:* 5′—CCG GAC GCT TAT GCC TTG CCA TCT ACA GAG CAG GTG TGA CGG—3′ *S. Typh*: 5′—ACG GGC GTG GGG GCA ATG CTG CTT GTA GCC TTC CCC TGT GCG CG—3′	[110]
MCF-7, HL-60, K562 cells (NR)	star-shaped/wax printing	Whatman chromatography paper No114	FRET	MCF-7: 5′-GCA GTT GAT CCT TTG GAT ACC CTG GTT TTT TTT TTT-NH_2_-3′ HL-60: 5′- NH2-TTT TTT TTT ATC CAG AGT GAC GCA GCA TGC CCT AGT TAC TAC TAC TCT TTT TAG CAA AC-3′ K562 cells: 5′- NH2-TTT TTT TTT TAC AGCAGA TCA GTC TAT CTT CTC CTG ATG GGT TCC TAT TTA TAG GTG AAG CTG T-3′	[111]
*Acinetobacter baumannii*, *Escherichia coli* and *Staphylococcus aureus*) (biological fluids)	microfluidic chip	nitrocellulose	colorimetric	A1, E27, O28	[112]

**Figure 15:**
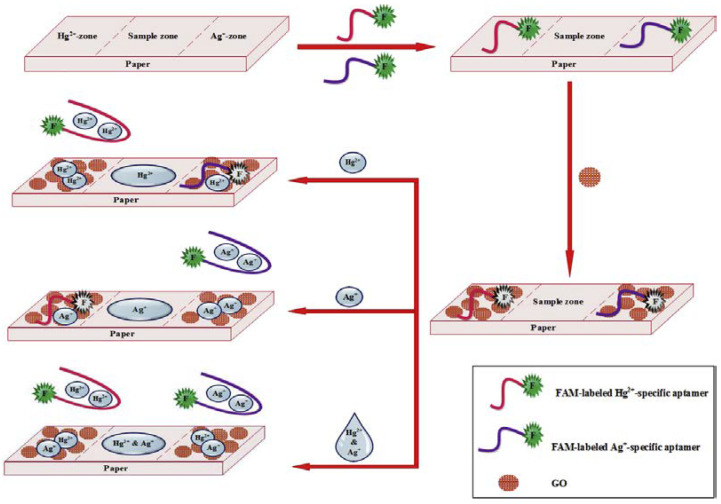
Illustration of the designed paper-based aptasensor for the simultaneous detection of Hg^2+^ and Ag^+^ (Reprinted with permission from Ref. [106]).

A low-cost paper-based aptasensor was developed featuring two test zones to simultaneously monitor Hg^2+^ and Ag^+^ using a FRET approach [106] (Figure 15). The linear ranges were 0.05–50 nM for both Hg^2+^ and Ag^+^ while the LODs for Hg^2+^ and Ag^+^ were 1.33 and 1.01 pM, respectively.

A paper-based microfluidic chip was developed in order to detect food allergens and food toxins (lysozyme, ß-conglutin, lupine, okadaic acid, and brevetoxin) simultaneously [107]. The targets were bound onto aptamer-functionalized quantum dots (QDs). After mixing with the GO, the fluorescence was quenched via the FRET process while the presence of target proteins restored the fluorescence intensity. The LODs ranged from 0.56 ng mL^−1^ to 343 ng mL^−1^, depending on the analyte.

**Figure 16:**
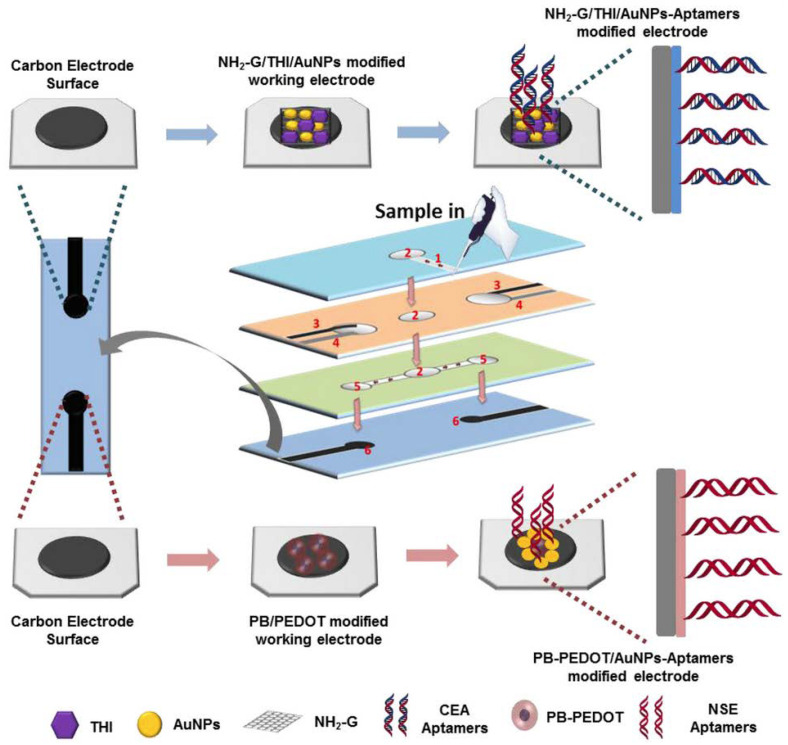
Fabrication and modification process of the multi-parameter electrochemical paper-based aptasensor for detection of carcinoembryonic antigen (CEA) and neuron-specific enolase (NSE): (1) sample inlet hole; (2) filter hole; (3) screen-printed counter electrode; (4) screen-printed reference electrode; (5) detection zone; (6) screen-printed working electrode (Reprinted with permission from Ref. [108]).

A duplex electrochemical aptasensor to detect simultaneously carcinoembryonic antigen and neuron-specific enolase has been reported [108] (Figure 16). The device makes use of a stacked microfluidic configuration. The working electrodes were modified with amino functional graphene/thionine/AuNPs and Prussian blue/poly (3, 4-ethylenedioxythiophene)/AuNPs nanocomposites to improve the electron transfer rate and immobilization efficiency of the aptamers. The aptasensor enables detection of carcinoembryonic antigen and neuron-specific enolase in ranges of 0.01–500 ng mL^−1^ and 0.05–500 ng mL^−1^, with LODs of 2 pg mL^−1^ and 10 pg mL^−1^, respectively.

An electrochemical lab-on-paper cyto-device was fabricated for specific cancer cell detection and in-situ monitoring of multi-glycans on cancer cells [109]. An aptamer-modified AuNPs-paper electrode was employed as the working electrode for cancer cell capture. Horseradish peroxidase-labeled with wheat germ agglutinin, peanut agglutinin, concanavalin A and with dolichos biflorus agglutinin were used as probes for the four glycans in a sandwich format. The device could detect the target cells in the range 550 to 2.0 × 10^7^ cells mL^−1^ and was applied to in-situ monitor cell-surface multi-glycans in parallel.

A paper-based microfluidic for duplex colorimetric detection of *E. coli O157:H7* and *S. Typhimurium* has been fabricated [110]. Polystyrene microparticles decorated with AuNPs were used as colorimetric labels for salt-based aggregation. Linearity held for 10^2^ CFU mL^−1^ to 10^8^ CFU mL^−1^ and the LODS were of 10^3^ CFU mL^−1^ and 10^2^ CFU mL^−1^ for *E. coli* O157:H7 and *S. Typhimurium*, respectively.

Liang et al. have developed a paper-based FRET aptasensing device for multiplexed monitoring of three kinds of cancer cells (MCF-7, HL-60, and K562) [111]. Quantum dots-coated silica nanoparticles are labeled with aptamers which are adsorbed on the surface of GO causing decrease in fluorescence; upon addition of the target cells, the fluorescence intensity is recovered. The linear ranges were from 180 to 8 × 10^7^, 210 to 7 × 10^7^, 200 to 7 × 10^7^ cells mL^−1^, and the LODs were 6270 and 65 cells mL^−1^ for MCF-7, HL-60, and K562 cells, respectively.

A microfluidic paper-based manifold has been designed for the multiplexed detection of three types of bacteria (*Acinetobacter baumannii*, *Escherichia coli,* and *Staphylococcus aureus*) [112]. Specific aptamers were immobilized on the nitrocellulose support and were used to capture the bacteria. Secondary biotin-labeled aptamers were incubated with the captured target bacteria, streptavidin-conjugated HRP was added and, finally, tetramethyl benzidine (TMB) reagent was used as a colorimetric probe. The LOD was ~10^3^ CFU μL^−1^ and the linear range ranged from 10^2^ to 10^5^ CFU μL^−1^.

## 6. Conclusions and Future Prospects

Aptamers have opened new directions for addressing some limitations of immunosensors, such as stability, cost, and scope for chemical functionalization, with the view to improve biosensor sensitivity, selectivity, and stability. However, despite having evolved as potential alternatives to antibodies, the market recognition of aptamers is low and the market share of aptasensor-based diagnostic products has not kept up with that of their antibody-based counterparts. This lag can be traced to some important technical issues related to aptamer-based technology: (a) the database of aptamers is still too small in comparison to the rich database of antibodies. The main reasons are the extensive usage of antibodies as capture probes for biosensor design for over 70 years and the relatively low screening efficiency and success rate of the conventional SELEX process which is also time-consuming and labor-intensive, (b) the structural flexibility of oligonucleotides means that many of the in vitro SELEX-selected aptamers in buffers may not retain their high affinity towards their targets under physiological conditions or in complex media, (c) aptamers are prone to nuclease degradation and their fast renal filtration may compromise their binding performance in in vivo imaging and sensing.

PADs have demonstrated wide potential for fulfilling critical demands for rapid and simple analytical testing in remote, resource-limited settings. Indeed, these properties have been largely responsible for the considerable recent interest and surge in research in the μPAD field. These devices provide a platform for a wide variety of (bio)chemical assays that can be employed to assess health issues, environmental pollution, and food quality. Yet, although many (nitro)cellulose-based devices (such as dipsticks, pH paper, and LFAs) have been successfully commercialized, there are hardly any commercial PADs on the market, despite some noteworthy promotion efforts such as Diagnostics for All (DFA) (http://dfa.org/, accessed on 9 August 2023) (a non-profit organization that aims to develop paper-based diagnostics for applications in resource-poor settings). Although PADs possess important characteristics including fabrication and operational simplicity, low cost, and portability that make them suitable for out-of-the-laboratory settings and in resource-limited environments, most of the current PAD implementations fail to fully comply with the WHO ASSURED criteria [113]. Among the main challenges to be tackled are [114]: (a) the low sensitivity often achieved with the widely used methods of colorimetric detection which can be potentially addressed by more sensitive detection techniques such as electrochemical and luminescence methods, (b) the poor reproducibility which is associated with the immobilization of reagents on the cellulose substrate, stability after long-term storage, evaporation issues and solution transport and mixing within the microfluidic conduit, all of which are difficult to control experimentally within resource-limited settings, (c) the inadequate specificity, expressed as false positives and, more critically as false negatives, (d) the low potential for multiplexed detection to test for several key markers, (e) the difficulty in data collection and objective interpretation.

There are several developments that are expected to facilitate the development of new aptasensing platforms and improve their performance including: (a) advances in the SELEX technology (Cell-SELEX, Slow off-rate modified aptamers (SOMAmers), microfluidic technologies, high-throughput sequencing (HTS) and parallel microarray characterization), (b) post-SELEX aptamer chemical modifications (PEGylation, sugar modification, phosphodiester linkage modification, and truncation), (c) in silico bioinformatics approaches (such as molecular dynamics simulations or molecular docking studies) applied during the selection and modification processes to estimate the aptamer-target binding affinity, to calculate thermodynamic and kinetic parameters and to optimize aptamer chemical modification, (d) exploitation of advantageous optical, electrical, or magnetic properties of various nanomaterials so that the sensitivity and specificity of such nanomaterial-based aptasensors could be significantly enhanced [5,43], (e) development of new antifouling agents to prevent aptasensor fouling due to matrix components in complex samples [115,116]. Regarding perspectives of PADs, the future trends include: (a) an increase in sensitivity through the exploitation of nanomaterials and new labeling reagents, optimization of the analytical protocol to maximize the aptamer-analyte interaction and use of more sensitive detection methods, (b) improving the physical arrangement of the devices using 3D or origami PADs, (c) exploiting the paper support for sample pre-treatment (e.g for filtration and preconcentration), (d) using more advanced fluid manipulation (mixing, flow delay, and solution switching), (e) increasing the multiplexing potential of the devices [117,118].

## Figures and Tables

**Table 1:** Examples of paper-based aptasensors for ions.

Analyte (Sample)	PAD	Type of Paper	Detection	Aptamer Sequence	Ref.
K^+^ (urine)	Circular-laser printing	Glossy paper	Colorimetric with AuNPs	5′-GGGTTAGGGTTAGGGTTAGGG-3′	[63]
Pb^2+^ (water)	Y-shaped fluidic-laser cutting	Whatman No 1, nylon	Colorimetric with AuNPs	5′-GGTTGGTGTGGTTGG-3′	[64]
Pb^2+^ (tap water, lake water, milk, blood serum)	Square-craft punch	Whatman No 1	FRET/GO	5′-FAMGGGTGGGTGGGTGGGT-3′	[65]
Hg^2+^ (water)	NR	Whatman No 1	CL	(S1): 5′-NH2-(CH2)6-CAGTTTGGAC-3′ (S2): 5′-NH2-GTCCTTTCTG-3′	[66]

## Data Availability

Not applicable.
